# ATF3 Promotes Arsenic-Induced Apoptosis and Oppositely Regulates DR5 and Bcl-xL Expression in Human Bronchial Epithelial Cells

**DOI:** 10.3390/ijms22084223

**Published:** 2021-04-19

**Authors:** Qiwen Shi, Bei Hu, Chen Yang, Lan Zhao, Jing Wu, Nan Qi

**Affiliations:** Collaborative Innovation Center of Yangtze River Delta Region Green Pharmaceuticals, Institute of Engineering Biology and Health, College of Pharmaceutical Sciences, Zhejiang University of Technology, Hangzhou 310014, China; qshi@zjut.edu.cn (Q.S.); hubei1994112@163.com (B.H.); 15271201029@163.com (C.Y.); 17326058655@163.com (L.Z.); JJWu1995@hotmail.com (J.W.)

**Keywords:** arsenic, ATF3, Bcl-xL, DR5, apoptosis

## Abstract

Arsenic is one of the most common environmental pollutants eliciting serious public health issues; however, it is also a well-recognized chemotherapeutic agent for acute promyelocytic leukemia. The association between arsenic exposure and lung diseases has been established, but underlying molecular mechanisms are poorly defined. Here we investigated the toxicology of arsenic in airway epithelium. Arsenic rapidly induced the activating transcription factor ATF3 expression through the JNK and p38 pathways. The ATF3-deficient BEAS-2B cells were relatively resistant to apoptosis upon arsenic exposure, indicating a facilitatory role of ATF3 in arsenic-induced apoptosis. We further showed that ATF3 oppositely regulated the transcription of death receptor (DR5) and Bcl2-like 1 (Bcl-xL) by directly binding to the promoter DR5 and Bcl-xL. Altogether, our findings establish ATF3 as a pro-apoptotic protein in arsenic-induced airway epithelial apoptosis through transcriptionally regulating DR5 and Bcl-xL, highlighting the potential of ATF3 as an early and sensitive biomarker for arsenic-caused lung injury.

## 1. Introduction

Arsenic is a metalloid element found mainly in water, air, soil and food, and it has been classified as a “group 1 carcinogen” by the International Agency for Research on Cancer (IARC). Exposure to arsenic has been associated with a variety of human diseases, such as cancer, peripheral neuropathy, peripheral vascular disorders and diabetes [1]. Environmental contamination and occupational exposure through oral and inhalation routes are the common ways of arsenic uptake in humans. As recommended by the World Health Organization (WHO, Geneva, Switzerland) and Environmental Protection Agency (EPA, Washington, DC, USA), the maximum concentration of arsenic in drinking water should not exceed 10 parts per billion (ppb) [2]. However, many areas in Bangladesh, China, Argentina, the United States and Canada have reported concentrations significantly higher than the limit, even hundreds of times [3]. The contamination of arsenic has been exacerbated not only due to global natural emission, but also human activities, including pesticides, combustion of fossil fuel and wastes from mining, and has become a major concern of public health globally that affects approximately 200 million of lives [2]. On the other hand, arsenic has been applied as a chemotherapeutic agent in clinic for acute promyelocytic leukemia (APL) [4]. 

The lung is the primary target of arsenic exposure. Both inhalation and ingestion of arsenic may lead to lung cancer and non-malignant lung diseases, such as chronic obstructive pulmonary disease (COPD) and impaired respiratory function [5]. The pathogenic effects of arsenic have been observed so far include reactive oxygen species (ROS) generation, DNA damage, epigenetic modification, mitochondrial dysfunction, autophagy dysfunction, glutathione depletion, proteotoxic stress, cell-cycle disruption and induction of aerobic glycolysis [6,7,8]. Genome-wide analysis of arsenic-exposed cells has revealed that arsenic prominently alters p53 signaling, oxidative stress and NF-κB signaling pathways [9]. Still, the underlying molecular mechanisms of arsenic-associated toxicity remains enigmatic. 

Activating transcription factor 3 (ATF3) belongs to the ATF/cyclic AMP response element-binding (CREB) family that binds to the consensus sequence TGACGTCA in promoters. As a stress-inducible gene, the basal level of ATF3 is low or even undetectable in most cells. Under physiological conditions, ATF3 expression can be rapidly induced by diverse stimuli like DNA damage agents, hypoxia, cytokines and chemokines. ATF3 then forms homodimers and heterodimers, which translocate into the nucleus to regulate the transcription of genes involved in cell cycle regulation, apoptosis, autophagy, DNA repair, ferroptosis, tumorigenesis and metastasis [10,11,12,13]. Moreover, ATF3 can regulate cellular functions independent of its transcriptional activity. For instance, ATF3 directly binds to Tip60 at a region adjacent to the catalytic domain, thereby enhancing the acetyltransferase activity and stability of Tip60 [14]. Many studies have identified ATF3 as a pro-apoptotic protein that results in cell death in response to cellular stresses, whereas it also has been shown that ATF3 protects cells from apoptosis by facilitating DNA lesions repair or removing DNA adducts [15]. The cell fate determined by ATF3 upregulation may be dependent on the p53 status, as UV-induced apoptosis is promoted by ATF3 in p53-defective cells, but the opposite outcome is observed in cells with normal p53 activity [15]. However, ATF3 silence reduces the rate of apoptosis induced by MX69 an inhibitor of MDM2/XIAP via suppressing p53 signaling in cholangiocarcinoma and is required for both the repair of ZnO nanoparticle (ZnO NP)-induced DNA damage and ZnO NP-induced apoptosis [16,17]. Therefore, the regulatory role of ATF3 in apoptosis and the downstream targets regulated by ATF3 are still in debate and need to be investigated. 

In this study, BEAS-2B cell line was used as the in vitro model to explore the toxicology of arsenic in the lung. Although p53 missense mutation occurred in BEAS-2B cells, the expression and responsiveness of p53 seem normal [18]. We report that ATF3 was upregulated by arsenic through the JNK and p38 pathways and promoted the arsenic-induced apoptosis in BEAS-2B cells. Arsenic-activated ATF3 enhanced death receptor 5 (DR5) expression and repressed Bcl2-like 1 (Bcl-xL) expression by directly binding to their promoter regions, thereby facilitating cell apoptosis through both intrinsic and extrinsic pathways. Our findings reveal the precise role of ATF3 in regulating apoptosis upon arsenic exposure and provide insights into the molecular mechanism of arsenic-associated toxicity.

## 2. Results

### 2.1. ATF3 Is Induced in BEAS-2B Cells upon Arsenic Exposure

**Figure 1:**
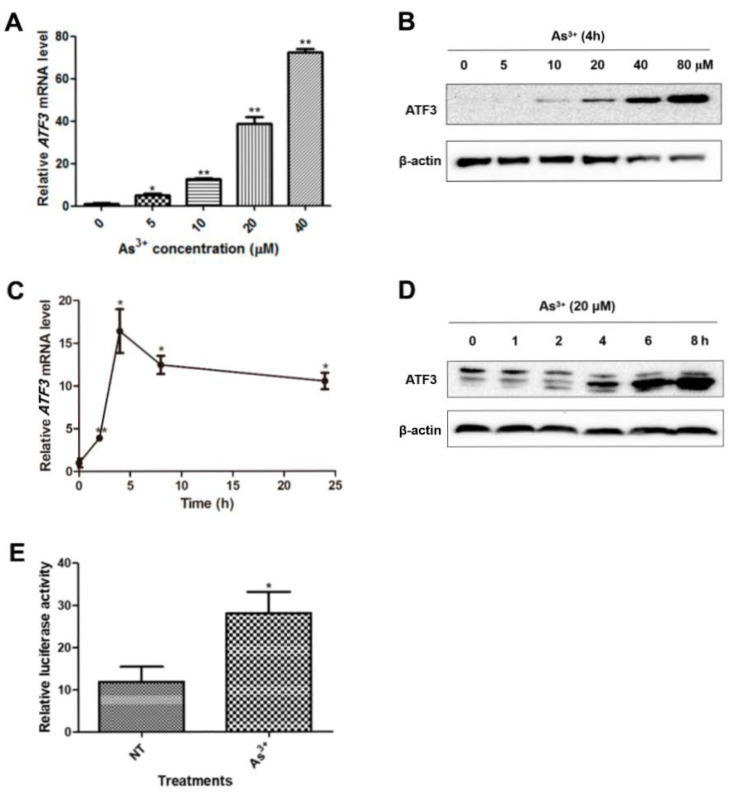
Arsenic induces ATF3 expression. (**A**,**B**) Total RNA and total protein were prepared from BEAS-2B cells exposed to arsenic with indicated concentrations for 4 h. ATF3 mRNA level was assessed by qRT-PCR, using GAPDH as the endogenous control, and ATF3 protein expression was detected by Western blot analysis. One-way ANOVA was performed for the statistics. (**C**,**D**) Cells were exposed to 20 µM of arsenic for indicated hours, and ATF3 mRNA and protein levels were measured. (**E**) Cells were co-transfected with pATF3-luc and pRL-TK and treated with or without arsenic for 16 h. The luciferase activity was measured. T-test was performed to analyze the statistical difference. All the data are presented as mean ± SD and represent three independent experiments. * *p* < 0.05 and ** *p* < 0.01, compared with non-treated cells.

ATF3 is known to be responsive to cellular stresses such as oxidative stress and DNA damage. Accordingly, we sought to determine whether the expression of ATF3 is altered upon arsenic exposure. We found that both the transcription and expression of ATF3 were significantly elevated by arsenic in BEAS-2B cells in a dose-dependent manner (Figure 1A,B). The increase of ATF3 mRNA level reached a peak at 4 h after arsenic treatment, while ATF3 protein expression continuously elevated during the 24 h arsenic exposure (Figure 1C,D). Consistently, the activity of ATF3 promoter, as validated by dual luciferase assay, was increased by approximately 2.4-fold in response to arsenic (Figure 1E). These results demonstrate that arsenic upregulates the ATF3 expression in BEAS-2B cells.

### 2.2. The Arsenic-Induced ATF3 Expression Relies on JNK and p38

**Figure 2:**
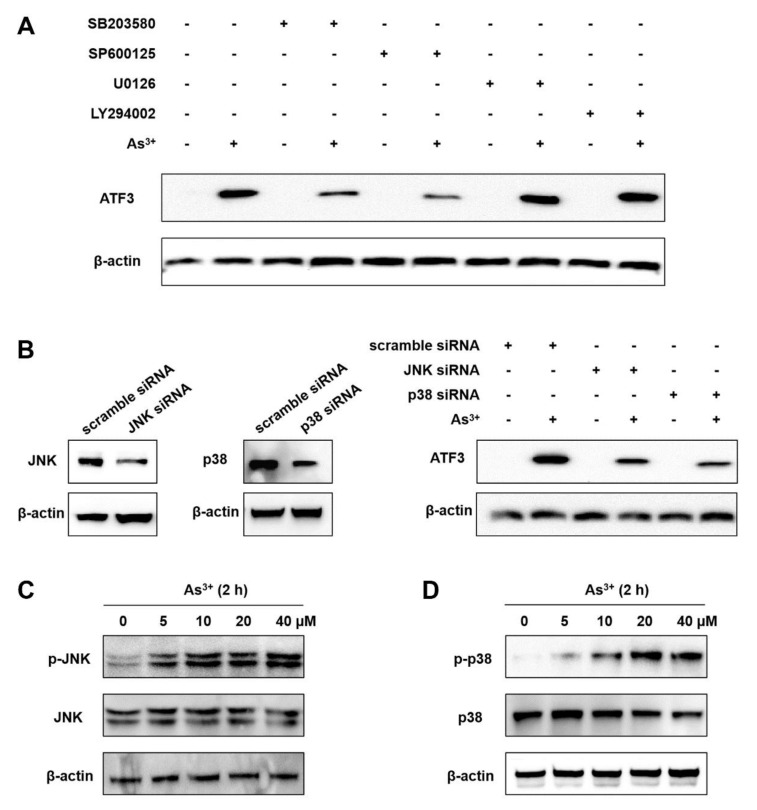
JNK and p38 activities are required for arsenic-induced ATF3. (**A**) BEAS-2B cells were pretreated with 10 μM SB203580 (p38 inhibitor), 10 μM LY294002 (PI3K inhibitor), 10 μM U0126 (ERK inhibitor) or 20 μM of SP600125 (JNK inhibitor) for 1 h, respectively, and then incubated with or without 20 μM arsenic for additional 4 h. Cells were lysed for Western blot analysis to measure ATF3 and β-actin (loading control) protein expression. (**B**) Cells were transfected with scramble siRNA, JNK siRNA or p38 siRNA and treated with or without 20 μM arsenic for 4 h. The expression of JNK, p38 and ATF3 was measured. (**C**,**D**) Cells were exposed to arsenic (0, 5, 10, 20 and 40 μM) for 2 h. The expression of p-JNK, JNK, p-p38, p38 and β-actin was measured.

Extracellular signal-regulated kinase (ERK), c-Jun N-terminal kinase (JNK) and p38 are three subfamilies that belong to mitogen-activated protein kinases (MAPKs) transducing extracellular signals into cells and involved in arsenic-related toxicity [19]. In addition, the PI3K/AKT pathway was reported to be strongly correlated with arsenic-induced cell proliferation, migration, invasion and anchorage-independent growth [3]. To determine whether the MPAK or PI3K/AKT pathways are involved in the arsenic-mediated ATF3 induction, BEAS-2B cells were pretreated with ERK, JNK, p38 or PI3K inhibitors for 1 h, respectively, followed by arsenic exposure. We observed that JNK inhibitor SP600125 and p38 inhibitor SB203580 dramatically reduced arsenic-upregulated ATF3 expression, whereas neither ERK inhibitor U0126 nor PI3K inhibitor LY294002 had any effects (Figure 2A). The inhibitory effect of U0126 and LY294002 on ERK and AKT phosphorylation, respectively, was confirmed and is shown in Supplementary Figure S1. Knockdown of JNK and p38 by siRNA interference also blocked arsenic-induced ATF3 expression (Figure 2B). Next, the phosphorylation of JNK and p38 was examined to confirm the activation of JNK and p38 pathways. Indeed, the levels of phosphorylated JNK and p38 were significantly increased at 2 h after arsenic exposure (Figure 2C,D). Thus, it is affirmative that the activities of JNK and p38 are indispensable for the arsenic-mediated ATF3 induction.

### 2.3. ATF3 Mediates the Arsenic-Induced Apoptosis

**Figure 3:**
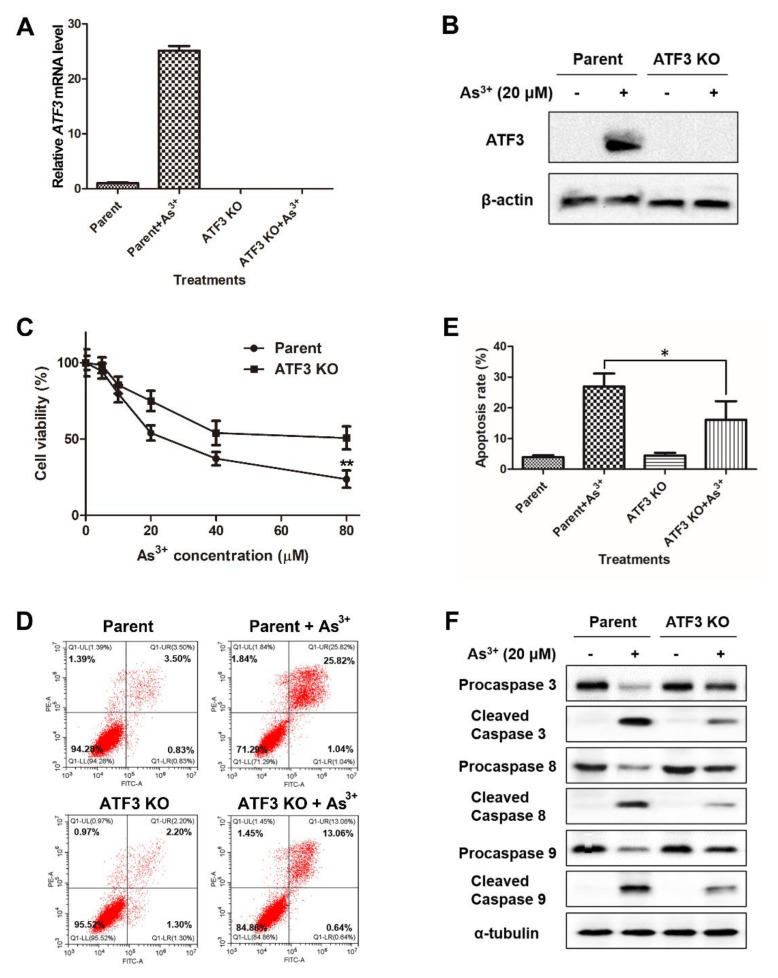
ATF3 functions as a pro-apoptotic protein in apoptosis induced by arsenic. (**A**,**B**) Parent and ATF3 KO cells were treated with or without 20 μM arsenic, and ATF3 mRNA level and protein expression were determined by qRT-PCR and Western blot analysis, respectively. (**C**) ATF3 KO and parent cells were exposed to arsenic (0, 5, 10, 20, 40 and 80 μM) for 24 h, and cell viability was measured by MTT assay. Data are presented as mean ± SD and represent three independent experiments. Statistics were performed by t-test; ** *p* < 0.01, compared with parent cells. (**D**) ATF3 KO and parent cells were treated with or without 20 μM arsenic for 24 h and then subjected to Annexin V-FITC/PI staining, which was analyzed by flow cytometry. (**E**) Percentage of apoptotic cells was the sum of percentages of early and late-phase apoptotic cells. Data are presented as mean ± SD of three independent experiments. Statistics were performed by paired t-test; * *p* < 0.05, compared with parent cells. (**F**) cell lysates were collected from ATF3 KO and parent cells treated with or without 20 μM arsenic for 24 h, and Western blot analysis was performed to detect the cleavage of caspase 3, 8 and 9.

To characterize the role of ATF3 in the arsenic-induced apoptosis, we employed CRISPR-Cas9 gene editing method to create the ATF3-deficient BEAS-2B cell line. The ATF3 deficiency was confirmed by qRT-PCR and Western blot analysis (Figure 3A,B). Next, both parent and ATF3 knockout (KO) cells were exposed to arsenic for 24 h, followed by various experiments to detect the levels of cell survival and apoptosis. Figure 3C shows that ATF3 KO cells were more resistant to arsenic-induced cell death than parent cells, as the cell viability was higher. Meanwhile, after arsenic treatment, the percentage of apoptotic cells was lower in ATF3 KO cells compared with parent cells (Figure 3D,E). Consistently, a smaller amount of procaspase 3, procaspase 8 and procaspase 9 was proteolytically cleaved into the active status in arsenic-exposed ATF3 KO cells compared with arsenic-exposed parent cells (Figure 3E). It is known that caspase 8 and caspase 9 are the initiators of extrinsic and intrinsic apoptosis, respectively, while caspase 3 is the executioner that ultimately elicits the morphological changes during apoptosis. Furthermore, overexpression of ATF3 in ATF3 KO cells restored the ability of cells to undergo arsenic-induced apoptosis (Supplementary Figure S2). Taken together, our data manifest that ATF3 contributes to both extrinsic and intrinsic apoptotic processes in BEAS-2B cells upon arsenic exposure.

### 2.4. Arsenic-Induced ATF3 Activates DR5 Transcription by Directly Binding to the DR5 Promoter

**Figure 4:**
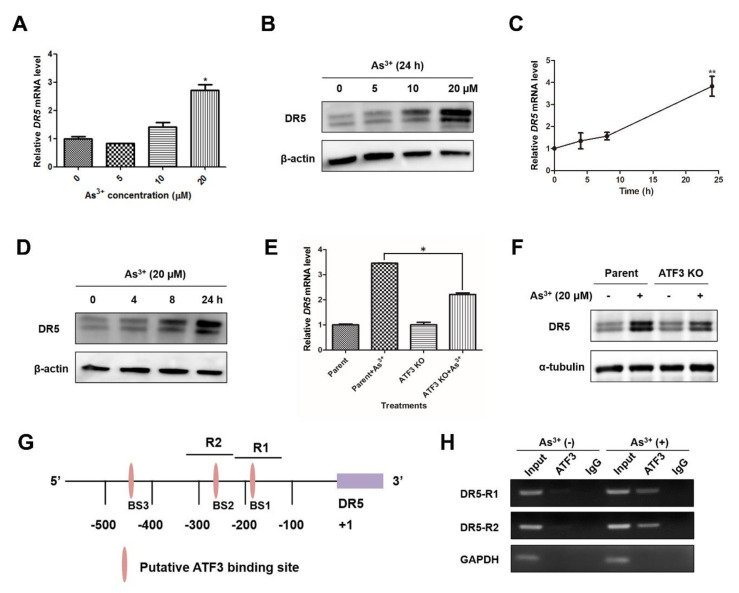
ATF3 directly binds to the DR5 promoter and enhances DR5 expression in response to arsenic. (**A**–**D**) Cells were exposed to arsenic (0, 5, 10 and 20 μM) for 24 h or to 20 μM arsenic for indicated hours. DR5 mRNA level and protein expression were analyzed by qRT-PCR and Western blot analysis, respectively. One-way ANOVA was performed for the statistics. (**E**,**F**) ATF3 KO and parent cells were treated with or without 20 μM arsenic for 24 h, and DR5 transcripts and protein were examined. (**G**) Schematic representation of the DR5 promoter. (**H**) BEAS-2B cells were treated with or without 20 μM arsenic for 12 h, and then ChIP assay was conducted. Data are presented as mean ± SD and represent three independent experiments. T-test was performed to determine the statistically significant difference from non-treated cells, or between arsenic-exposed parent and ATF3 KO cells. * *p* < 0.05 and ** *p* < 0.01.

DR5 is a member of the tumor necrosis factor (TNF) receptor family that is responsive to signals such as TNF, FasL and TNF-related apoptosis-inducing ligand (TRAIL). The activation of DR5 recruits the adaptor protein Fas-associated protein with death domain (FADD) and caspase 8 or 10 to form death-inducing signal complex (DISC), and subsequently triggers extrinsic pathway of apoptosis [20]. Meanwhile, DR5 can cause the interaction among Bid, Bax and Bak by caspase 8-mediated cleavage of Bid, resulting in the activation of intrinsic apoptosis [20]. Previous studies have indicated that ATF3 can upregulate DR5 expression in the context of anti-cancer agents camptothecin (CPT), celecoxib and zerumbone, as well as environmental carcinogen tetrachlorobenzoquinone (TCBQ) [20,21,22]. Here, we hypothesized that ATF3 participates the arsenic-induced apoptosis through activating DR5. In the context of arsenic, a dose- and time-dependent elevation of DR5 mRNA level and protein expression was confirmed by qRT-PCR and Western blot analysis (Figure 4A–D). Next, ATF3 KO and parent cells were exposed to 20 μM arsenic to examine the role of ATF3 in arsenic-upregulated DR5 expression. As shown in Figure 4E,F, the ATF3-deficiency significantly repressed the induction of DR5 mRNA and protein under arsenic treatment, compared with parent cells. There are four putative ATF/CRE motifs in the human DR5 promoter region, whereas only the two binding sites proximal to the transcription start site, which are labeled as BS1 and BS2 in Figure 4G, are fully required for DR5 induction [20,21]. To verify whether the arsenic-induced ATF3 upregulates DR5 expression by directly binding to the DR5 promoter, we conducted ChIP assay, targeting the two promoter motifs essential for DR5 induction, termed as R1 and R2 (Figure 4G). Upon arsenic exposure, ATF3 protein was indeed recruited onto the DR5 promoter, as a significant amplification of regions R1 and R2 was obtained in arsenic-treated cells by immunoprecipitation with anti-ATF3 antibody (Figure 4H). No bands were observed in the same ChIP assay performed in ATF3 KO cells (Supplementary Figure S3). Collectively, these data reveal that ATF3 binds to the ATF/CRE motifs in the promoter region of DR5, thereby activating DR5 expression in response to arsenic exposure.

### 2.5. ATF3 Is Recruited onto the Bcl-xL Promoter and Represses Bcl-xL Expression upon Arsenic Exposure

**Figure 5:**
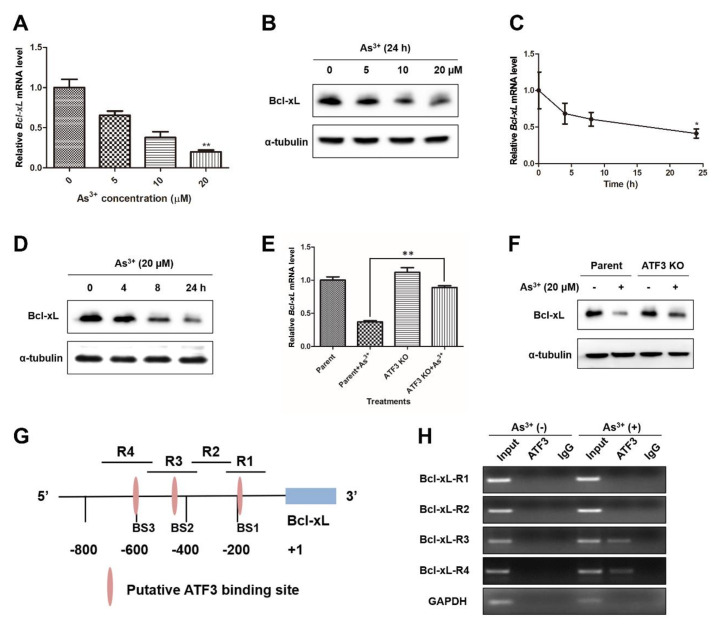
ATF3 is recruited onto the Bcl-xL promoter and represses Bcl-xL expression upon arsenic insult. (**A**–**D**) Cells were exposed to arsenic (0, 5, 10 and 20 μM) for 24 h or to 20 μM arsenic for indicated hours, and subjected to qRT-PCR and Western blot analysis for Bcl-xL expression, respectively. One-way ANOVA was performed for the statistics. (**E**,**F**) ATF3 KO and parent cells were treated with or without 20 μM arsenic for 24 h, and Bcl-xL mRNA and protein levels were determined. (**G**) The putative ATF3 binding sites on the Bcl-xL promoter is shown. (**H**) BEAS-2B cells treated with or without arsenic were subjected to ChIP assays with an anti-ATF3 antibody, and the amount of precipitated DNA was measured by PCR. Data are presented as mean ± SD and represent three independent experiments. T-test was conducted to determine the statistically significant difference from non-treated cells, or between arsenic-exposed parent and ATF3 KO cells. * *p* < 0.05 and ** *p* < 0.01.

Bcl-xL is a pro-survival protein that inhibits apoptosis by preventing the disruptive effect of Bax on outer mitochondrial membrane integrity and disturbing DISC formation [23]. The negative regulation of ATF3 on Bcl-xL makes cancer cells more sensitive to histone deacetylase inhibitors (HDACi) [24]. To see if ATF3 mediates the arsenic-induced apoptosis through the repression of Bcl-xL, we examined the expression of Bcl-xL upon arsenic exposure. Both Bcl-xL mRNA level and protein expression were decreased dose- and time-dependently when BEAS-2B cells were treated with arsenic (Figure 5A–D). ATF3 KO cells were more resistant to the reduction of Bcl-xL expression during arsenic treatment in comparison with parent cells, indicating the requirement of ATF3 in arsenic-mediated repression of Bcl-xL (Figure 5E,F). Furthermore, we performed ChIP assay to find out whether ATF3 directly binds to the Bcl-xL promoter to suppress Bcl-xL transcription. According to previous analysis, there are three putative ATF/CRE motifs and an AP-1 site on the Bcl-xL promoter (Figure 5G) [24]. The enrichment of ATF3 binding following arsenic treatment was noticed at regions R3 and R4, indicating the recruitment of ATF3 onto the binding sites BS2 and BS3 on the Bcl-xL promoter. No binding of ATF3 at BS2 or BS3 was detected in ATF3 KO cells exposed to arsenic (Supplementary Figure S4). Altogether, our evidence proves that ATF3 suppresses Bcl-xL expression in response to arsenic exposure by direct binding to the ATF/CRE binding sites on the Bcl-xL promoter.

## 3. Discussion

**Figure 6:**
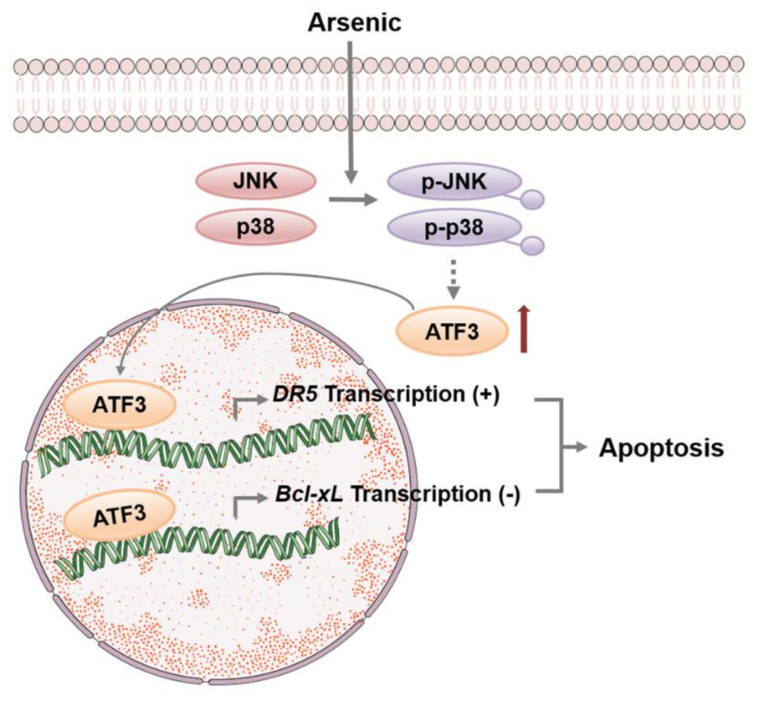
A schematic model of the regulation of ATF3 on DR5 and Bcl-xL expression upon arsenic exposure. Arsenic induces ATF3 through JNK and p38, and then ATF3 is recruited onto the promoter regions of DR5 and Bcl-xL to enhance DR5 expression and repress Bcl-xL expression. The opposite regulation of ATF3 on DR5 and Bcl-xL expression facilitates arsenic-induced apoptosis.

ATF3 has been shown to be responsive to various stresses and determine cell fate by either mediating apoptosis or facilitating DNA repair. In this study, we clearly demonstrated that, in BEAS-2B cells, ATF3 is induced upon arsenic exposure in a JNK- and p38-dependent manner. Arsenic-induced ATF3 then translocates into the nucleus and directly binds to the promoter regions of DR5 and Bcl-xL to oppositely regulate the expression of DR5 and Bcl-xL. The enhanced-expression of DR5 and suppressed-expression of Bcl-xL mediated by ATF3 contribute to arsenic-induced apoptosis, indicating that ATF3 acts as a pro-apoptotic transcription factor in arsenic-associated cytotoxicity (Figure 6). 

Although the basis for lung selectivity of arsenic exposure is not understood yet, the accumulation of arsenic in lung has been observed during chronic and high-level arsenic exposure [25]. Epidemiological studies have revealed a high correlation between arsenic pollution and pulmonary disorders such as increased airway inflammation, the incidence of lung cancer and restrictive pattern lung diseases [26,27,28]. In addition, in vivo experiments have manifested that oral administration of arsenic disrupts airway immune responses and redox status in mice [29,30]. BEAS-2B cells are primary normal human bronchial epithelial cells immortalized by SV40 and have been identified as a classic in vitro model to study the toxicology of arsenic in airway epithelium. Therefore, BEAS-2B cells were used in our study to explore the molecular mechanisms of arsenic-induced cytotoxic effects in lung. 

It was reported that ATF3 induction is mediated by JNK and p38 in Streptococcus Pneumoniae-infected murine macrophages [31]. However, the involvement of MAPK subfamilies in ATF3 upregulation varies depending on stimuli. For example, p38 is necessary for the induction of ATF3 by anisomycin, inflammatory cytokines and H_2_O_2_, whereas neither ERK nor JNK participates in anisomycin-induced ATF3 [32]. Besides MAPK signaling, ATF3 induction under different pathological conditions may rely on the activation of other pathways such as JAK/STAT, SMAD and c-myc [10]. In the present work, we observed the increased phosphorylation of JNK and p38 by arsenic and addressed the indispensability of JNK and p38 activation for arsenic-induced ATF3. Our findings thus indicate that JNK and p38 pathways rather than ERK or PI3K/Akt pathways are the upstream regulators of ATF3 expression induced by arsenic.

Our study reported that arsenic triggered both intrinsic and extrinsic apoptosis in BEAS-2B cells, and the apoptosis was compromised in ATF3-deficient cells. During the investigation of potential mechanisms, we found that ATF3-deficient cells expressed a lower level of DR5 and a higher level of Bcl-xL upon arsenic exposure compared with parent cells, and the regulation of ATF3 on DR5 and Bcl-xL was at transcriptional level, since ATF3 was able to bind to the promoter of DR5 and Bcl-xL directly. In many cancers, ATF3 expression is enhanced by anti-tumor agents and has positive effects on apoptosis. For instance, withaferin A, a bioactive compound derived from Withania somnifera, activates the ATF4–ATF3–CHOP axis to initiate apoptosis in glioblastoma cells [33]. In NSCLC cells, pemetrexed, a folate antimetabolite, induces apoptosis through the ATF4-ATF3–Noxa–Usp9x–Mcl-1 pathway [34]. It would be intriguing to test whether the identified downstream targets related to the pro-apoptotic activity of ATF3 are universally or cell- and stimulus-specific.

Previous research suggests that ATF3 homodimers repress gene expression and heterodimers (with other bZip proteins) activate or inhibit transcription [10]. This description implies that the gene expression regulated by ATF3 and the exact regulation of ATF3 on transcription may depend on the protein interacted with ATF3. For example, overexpression of ATF3 represses PTEN-induced putative kinase 1 (PINK1) gene transcription in lung epithelial cells, while ATF3/c-Jun complex stimulates cannabinoid receptor type 1 (CB1R) transcription in chronic kidney disease (CKD) [35,36]. Therefore, further elucidation for the mechanism by which ATF3 regulates transcription is required.

Our research indicates that ATF3 is an early and sensitive biomarker for arsenic exposure, with the observation that ATF3 mRNA level and protein expression were elevated significantly at 2 and 4 h after arsenic exposure, respectively. Herein, ATF3 seems to be a good indicator for early lung injury caused by high-dose arsenic intoxication. As is well-known, high concentrations of arsenic can elicit rapid toxic effects leading to death, while low concentrations of arsenic are unlethal but threaten human health through the potent carcinogenic effects. Meanwhile, low-dose and high-dose arsenic exposure may trigger different cellular possesses. Therefore, our next step is to investigate the role of ATF3 in arsenic-induced carcinogenesis and the molecular mechanisms, and to determine whether ATF3 is an attractive target to alleviate arsenic-caused lung injury or prevent arsenic-induced carcinogenesis. Moreover, given that ATF3 promotes the arsenic-induced apoptosis, targeting ATF3 may have synergic effects with arsenic in APL treatment.

## 4. Materials and Methods

### 4.1. Cell Culture and Reagents

Human bronchial epithelial BEAS-2B cells were purchased from the China Center for Type Culture Collection (CCTCC, Wuhan, China) and cultured in Dulbecco’s Modified Eagle Medium (DMEM, Invitrogen, San Diego, CA, USA) supplemented with 10% fetal bovine serum (FBS, Gibco, NY, USA). ATF3 KO BEAS-2B cells were custom developed by using a CRISPR/Cas9 gene-editing method by Cyagen Biosciences (Suzhou, China). Sodium meta-arsenite (NaAsO_2_) was purchased from Sigma (St Louis, MO, USA). The inhibitors, SB203580, SP600125, U0126 and LY294002, were purchased from Santa Cruz Biotechnology (Dallas, TX, USA). Antibodies against ATF3 (33593), DR5 (3696), JNK (9252), p-JNK (4668), p38 (8690), p-p38 (9215), Bcl-xL (2764), caspase 8 (9746), cleaved caspase 8 (8592), caspase 3 (14220), cleaved caspase 3 (9664), caspase 9 (9502), cleaved caspase 9 (9509), α-tubulin (2125), AKT (C67E7), p-AKT (4060T), ERK (4695), p-ERK (91015) and β-actin (4970) were purchased from Cell Signaling Technologies (Danvers, MA, USA).

### 4.2. Plasmid Construct and Dual Luciferase Assay

The human ATF3 promoter was synthesized by GenScript Biotech Corporation (Nanjing, China), and subcloned into pGL3 vector to construct pATF3-luc that expresses Firefly luciferase under the control of ATF3 promoter. BEAS-2B cells were transfected with pATF3-luc and a plasmid constitutively expressing Renilla luciferase (pRL-TK) by lipofectamineTM 3000 (Invitrogen, San Diego, CA, USA). Cells were treated with or without arsenic and processed by using Dual-luciferase Reporter Assay System (Promega, Madison, WI, USA) as per the manufacturer’s instruction. Firefly luciferase expression was normalized to Renilla luciferase expression.

### 4.3. RNA Isolation and Quantitative RT-PCR

The total RNA was extracted by using a GeneJet RNA purification kit (Thermo Fisher Scientific, Inc., Waltham, MA, USA), and reverse transcription was conducted by using a cDNA Synthesis Kit (Thermo Fisher Scientific, Inc., Waltham, MA, USA). Then qPCR was performed as described previously [37]. In this study, the following primer pairs were used: ATF3, 5′-CCTCTGCGCTGGAATCAGTC-3′ (forward) and 5′-TTCTTTCTCGTCGCCTCTTTTT-3′(reverse); DR5, 5′-ACAGTTGCAGCCGTAGTCTTG-3′ (forward) and 5′-CCAGGTCGTTGTGAGCTTCT-3′ (reverse); Bcl-xL, 5′-GAGCTGGTGGTTGACTTTCTC-3′ (forward) and 5′-TCCATCTCCGATTCAGTCCCT-3′ (reverse); GAPDH, 5′-GGAGCGAGATCCCTCCAAAAT-3′ (forward) and 5′-GGCTGTTGTCATACTTCTCATGG-3′ (reverse).

### 4.4. Protein Isolation and Western Blot Analysis

Whole cell extracts were prepared for analysis by Western blotting as described in a previous study [38]. Protein lysates were separated by SDS-PAGE and transferred onto PVDF membranes by electrophoretic transfer. The blots were blocked in TBST containing 5% fat free milk and incubated with a primary antibody at 4 °C, overnight. Then the blots were washed with TBST and incubated with horseradish peroxidase-conjugated anti-mouse/rabbit antibody, at room temperature, for 1 h. The bound antibodies were detected by Super Signal West Pico Kit (Thermo Fisher Scientific, Inc., Waltham, MA, USA).

### 4.5. Apoptosis Analysis by Flow Cytometry

Parent or ATF3 KO BEAS-2B cells were seeded in 6-well plates and treated with or without 20 μM arsenic for 24 h. Then cells were detached by trypsin and stained by using Annexin V-FITC Apoptosis Detection Kit (BD Bioscience, Franklin Lakes, NJ, USA) according to manufacturer’s instruction. Apoptotic cells were measured by Beckman CytoFLEX S (Beckman Coulter, Brea, CA, USA).

### 4.6. Cell Viability Assay

Parent or ATF3 KO BEAS-2B cells were placed in a 96-well plate, at a density of 5 × 10^3^ cells/well, and exposed to different concentrations of arsenic. Then, 24 h later, cells were stained with MTT for 4 h. The medium was then removed, and formazan crystals were dissolved by DMSO. Absorbance was measured at 570 nm, using a microplate reader.

### 4.7. Chromatin Immunoprecipitation (ChIP) Assay

ChIP assay was performed using SimpleChIP Enzymatic Chromatin IP kit (Cell Signaling Technology, Danvers, MA, USA). Briefly, arsenic-exposed or non-exposed BEAS-2B cells were treated with 1% formaldehyde for 10 min. After enzymatic reaction, immunoprecipitation was performed at 4 °C, overnight, with rotation, using anti-ATF3 antibody or control IgG. A small chromatin-protein sample was kept before immunoprecipitation and used as input. Reverse-crosslink was conducted at 65 °C for 2 h, and then DNA was purified. PCR was performed using the following primers: DR5-R1, 5′-GCAGTTGCACATTGGATCTG-3′ (forward) and 5′-TATGTGTCCAGGCTGACTTG-3′ (reverse); DR5-R2, 5′-AAGGTTAGTTCCGGTCCCTTC-3′ (forward) and 5′-TTCCACCACAGGTTGGTGAC-3′ (reverse); Bcl-xL-R1, 5′-GAAACCTTGAACCCCATTGA-3′ (forward) and 5′-GGCTCTCCGCCTCCTACT-3′ (reverse); Bcl-xL-R2, 5′-CTTTGGGGAATTCAGAGCAA-3′ (forward) and 5′-GGACTTCTCAATGGGGTTCA-3′ (reverse); Bcl-xL-R3, 5′-CAGGAAAACGTGGTCTCAGC-3′ (forward) and 5′-TTGCTCTGAATTCCCCAAAG-3′ (reverse); Bcl-xL-R4, 5′-CCCTCCTCTCAGGAAGGTCT-3′ (forward) and 5′-CAGCTGAGACCACGTTTTCC-3′ (reverse); GAPDH, 5′-CTTGACTCCCTAGTGTCCTTC-3′ (forward) and 5′-AAGGTCTTGAGGCCT-3′ (reverse).

### 4.8. Transfection of siRNA and Plasmids

The JNK siRNA: 5′-CCUACCUUCUCUAUCAGAUTT-3′ (sense); 5′-AUCUGAUAGAGAAGGUAGGTT-3′ (antisense). The p38 siRNA: 5′-GGACUAUUUAUUCCAGCUUTT-3′ (sense); 5′-AAGCUGGAAUAAAUAGUCCTT-3′ (antisense). ATF3 cDNA was synthesized by GenScript (Nanjing, China) and inserted into pcDNA3.1. Transfection was performed by using Lipofectamine 3000.

### 4.9. Statistical Analysis

Data are presented as mean ± SD. Two-tailed *t*-test was used for a comparison of two datasets. One-way ANOVA was used for a comparison of more than two groups. A *p* value < 0.05 was considered statistically significant.

## Data Availability

The data presented in this study are available on request from the corresponding author.

## Supplementary Materials

The following are available online at https://www.mdpi.com/article/10.3390/ijms22084223/s1, Figure S1: The inhibition of U0126 and LY294002 on ERK and AKT pathway, respectively. Figure S2: Overexpression ATF3 in ATF3 KO BEAS-2B cells restored arsenic-induced apoptosis. Figure S3: ChIP assay with ATF3 KO BEAS-2B cells. Figure S4: Using ATF3 KO BEAS-2B cells for ChIP assay as negative control.
