# Correction: Shi et al. ATF3 Promotes Arsenic-Induced Apoptosis and Oppositely Regulates DR5 and Bcl-xL Expression in Human Bronchial Epithelial Cells. *Int. J. Mol. Sci.* 2021, *22*, 4223

**DOI:** 10.3390/ijms26189046

**Published:** 2025-09-17

**Authors:** Qiwen Shi, Bei Hu, Chen Yang, Lan Zhao, Jing Wu, Nan Qi

**Affiliations:** Collaborative Innovation Center of Yangtze River Delta Region Green Pharmaceuticals, Institute of Engineering Biology and Health, College of Pharmaceutical Sciences, Zhejiang University of Technology, Hangzhou 310014, China

In the original publication [1], there was a mistake in Figure 1 as published. Unintentionally, the representative images of β-actin were published in a previous study conducted by the first author [2], because the proteins (ATF3 and Egr-1) investigated in these two projects were detected on the same blot and shared the same loading control. Moreover, the last time point was mislabeled in Figure 1D. The corrected Figure 1 appears below. The authors state that the scientific conclusions are unaffected. This correction was approved by the Academic Editor. The original publication has also been updated.

## Figures and Tables

**Figure 1:**
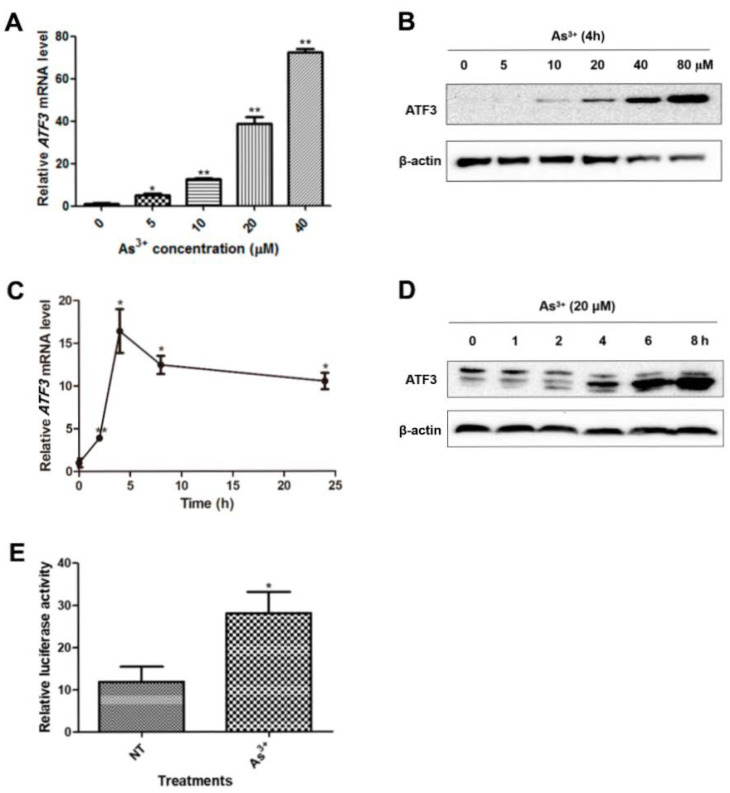
Arsenic induces ATF3 expression. (**A**,**B**) Total RNA and total protein were prepared from BEAS-2B cells exposed to arsenic with indicated concentrations for 4 h. ATF3 mRNA level was assessed by qRT-PCR, using GAPDH as the endogenous control, and ATF3 protein expression was detected by Western blot analysis. One-way ANOVA was performed for the statistics. (**C**,**D**) Cells were exposed to 20 µM of arsenic for indicated hours, and ATF3 mRNA and protein levels were measured. (**E**) Cells were co-transfected with pATF3-luc and pRL-TK and treated with or without arsenic for 16 h. The luciferase activity was measured. T-test was performed to analyze the statistical difference. All the data are presented as mean ± SD and represent three independent experiments. * *p* < 0.05 and ** *p* < 0.01, compared with non-treated cells.
