# Supplementary material for: ATF3 Promotes Arsenic-Induced Apoptosis and Oppositely Regulates DR5 and Bcl-xL Expression in Human Bronchial Epithelial Cells
Source: Int J Mol Sci. 2021 Apr 19;22(8):4223. doi: 10.3390/ijms22084223 (PMC8072958; doi:10.3390/ijms22084223)

Supplementary Figure 1

The inhibition of U0126 and LY294002 on ERK and AKT pathway, respectively.

(A) BEAS-2B cells were pretreated with 10 μM U0126 for 1 h and then exposed to 20 μM arsenic for 4 h. The expression of p-ERK and ERK was measured by western blot to confirm the inhibitory effect of U0126.

(B) BEAS-2B cells were pretreated with 10 μM LY294002 for 1 h and then exposed to 20 μM arsenic for 4 h. The expression of p-AKT and AKT was measured by western blot to confirm the inhibitory effect of LY294002.

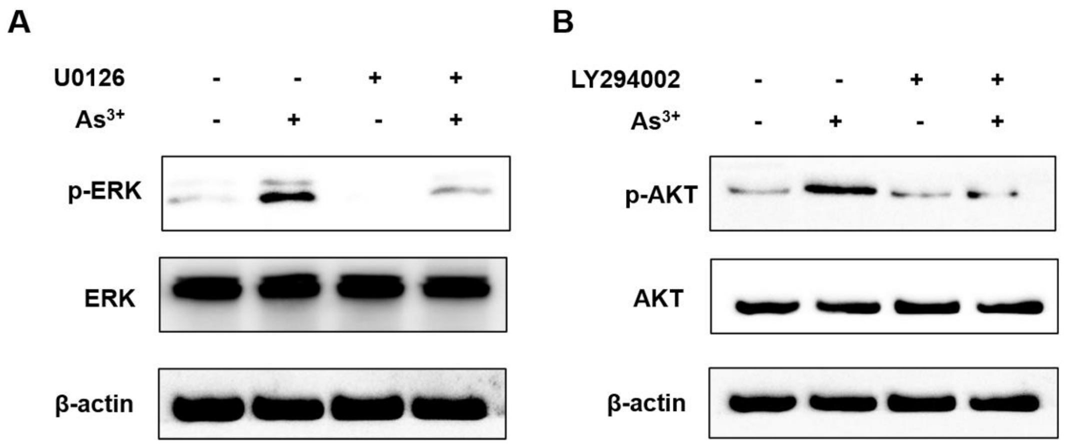

Supplementary Figure 2

Overexpression ATF3 in ATF3 KO BEAS-2B cells restored arsenic-induced apoptosis.

ATF3 KO BEAS-2B cells were transfected with empty vector or ATF3-expressing plasmid (pATF3), followed by exposure to 20 μM arsenic for 24 h.

(A) The overexpression of ATF3 was confirmed by western blot.

(B) The expression of procaspase 3 and cleaved caspase 3 was detected by western blot.

(C) Cells were stained with Annexin V/PI, and examined by flow cytometry.

(D) Percentage of apoptotic cells was the sum of percentages of early- and late-phase apoptotic cells from the results obtained by flow cytometry. Data are presented as mean ± SD of three independent experiments. Statistics was performed by paired t-test. * $p < 0.05$, compared with parent cells.

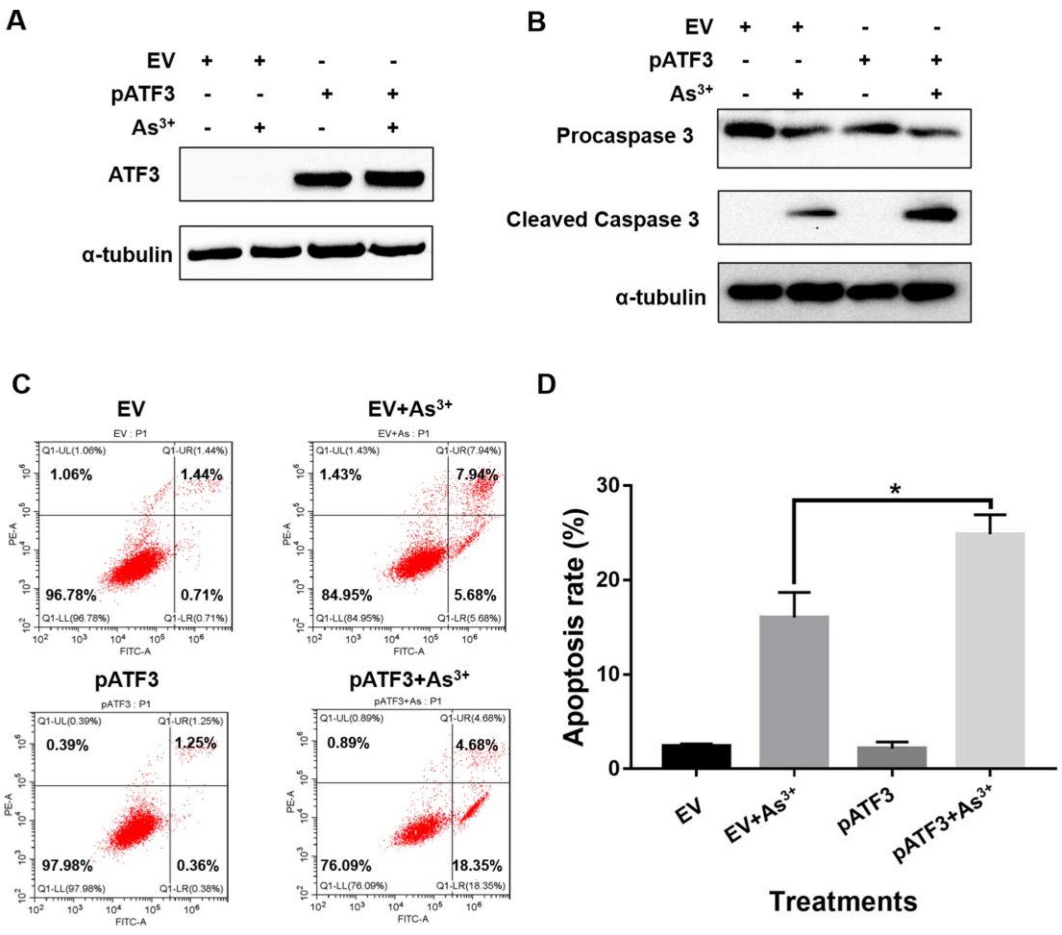

Supplementary Figure 3

ChIP assay with ATF3 KO BEAS-2B cells.

ATF3 KO BEAS-2B cells were treated with or without 20 μm arsenic for 12 h. ChIP assay was conducted.

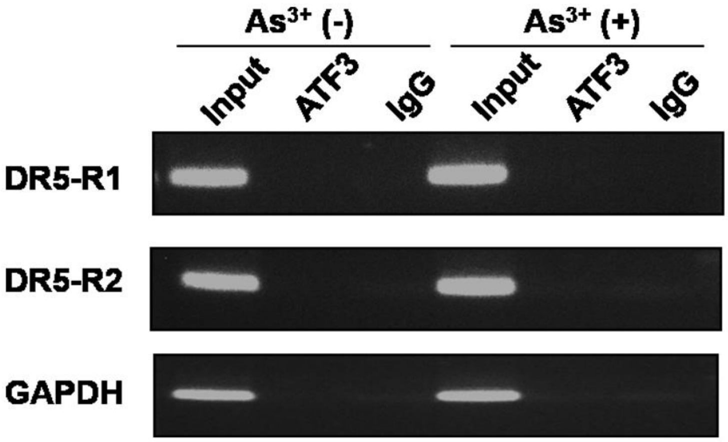

Supplementary Figure 4

Using ATF3 KO BEAS-2B cells for ChIP assay as negative control.

ATF3 KO BEAS-2B cells were treated with or without 20 μm arsenic for 12 h. ChIP assay was conducted.

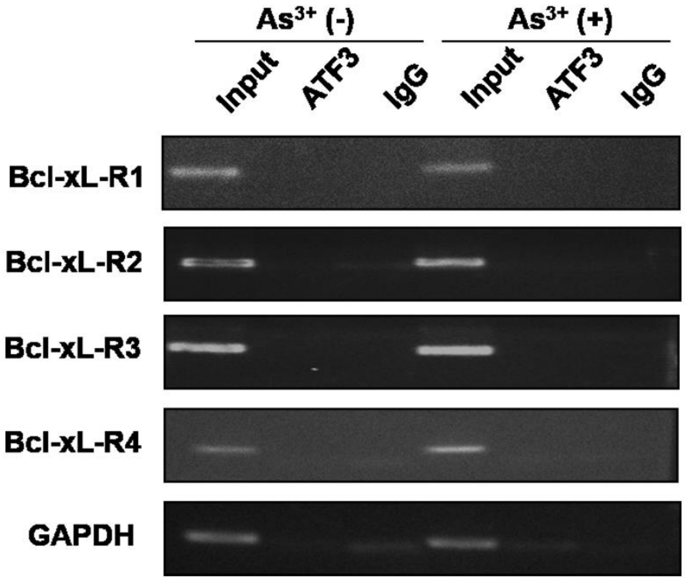

Supplement: Supplementary file 1 [file ijms-22-04223-s001.zip › ijms-1160456-supplementary.pdf]
